# Ultra-Soft PDMS-Based Magnetoactive Elastomers as Dynamic Cell Culture Substrata

**DOI:** 10.1371/journal.pone.0076196

**Published:** 2013-10-18

**Authors:** Matthias Mayer, Raman Rabindranath, Juliane Börner, Eva Hörner, Alexander Bentz, Josefina Salgado, Hong Han, Holger Böse, Jörn Probst, Mikhail Shamonin, Gareth J. Monkman, Günther Schlunck

**Affiliations:** 1 Department of Electrical Engineering and Information Technology, Regensburg University of Applied Sciences, Regensburg, Germany; 2 Center Smart Materials, Fraunhofer Institut für Silicatforschung, Würzburg, Germany; 3 Department of Ophthalmology, Würzburg University Hospital, Würzburg, Germany; 4 Department of Ophthalmology, Freiburg University Medical Center, Freiburg, Germany; Faculdade de Medicina Dentária, Universidade do Porto, Portugal

## Abstract

Mechanical cues such as extracellular matrix stiffness and movement have a major impact on cell differentiation and function. To replicate these biological features in vitro, soft substrata with tunable elasticity and the possibility for controlled surface translocation are desirable. Here we report on the use of ultra-soft (Young’s modulus <100 kPa) PDMS-based magnetoactive elastomers (MAE) as suitable cell culture substrata. Soft non-viscous PDMS (<18 kPa) is produced using a modified extended crosslinker. MAEs are generated by embedding magnetic microparticles into a soft PDMS matrix. Both substrata yield an elasticity-dependent (14 vs. 100 kPa) modulation of α-smooth muscle actin expression in primary human fibroblasts. To allow for static or dynamic control of MAE material properties, we devise low magnetic field (≈40 mT) stimulation systems compatible with cell-culture environments. Magnetic field-instigated stiffening (14 to 200 kPa) of soft MAE enhances the spreading of primary human fibroblasts and decreases PAX-7 transcription in human mesenchymal stem cells. Pulsatile MAE movements are generated using oscillating magnetic fields and are well tolerated by adherent human fibroblasts. This MAE system provides spatial and temporal control of substratum material characteristics and permits novel designs when used as dynamic cell culture substrata or cell culture-coated actuator in tissue engineering applications or biomedical devices.

## Introduction

Most cells transform mechanical stimuli into intracellular signals in a process termed mechanotransduction [1]. Based on this principle, biomechanical cues such as extracellular matrix (ECM) strain and elasticity have a decisive influence on cell differentiation and function [2], [3], [4] and altered tissue biomechanics appear to play a role in several diseases such as atherosclerosis or cancer [5], [6]. Cells reside in a soft ECM microenvironment in vivo (Young’s modulus ∼10^0^–10^2^ kPa) [3] whose elastic properties are not comparable to standard polystyrene cell culture substrata. To adequately reproduce biomechanical tissue properties in vitro, soft articulated cell culture substrata are desirable. Polyacrylamide (PA) hydrogels were used in seminal experiments to provide a 2D cell culture microenvironment of suitable elasticity [3], [7] and to decouple effects of ligand density and other mechanical properties. To date, several polymer hydrogel materials have been used in cell culture applications. However, the current need for laborious customized gel preparation serves to impede the general usage of hydrogels as standard cell culture substrata. Care must be taken to eliminate toxic unlinked monomers following gel preparation and gel swelling must be considered when changing media. Furthermore, the hydrogel fluid space communicates with supernatant media and influences its composition in a manner which may be difficult to control. Gel actuation may induce fluid shifts and concomitant shear forces with adverse effects on the attached cells. Magnetoactive hydrogels have recently been developed for biomedical use and hold promise as tissue engineering scaffolds, drug delivery systems and localized hyperthermia generators for cancer treatment [8], [9]. Elastic PDMS-based cell culture substrata may offer advantages over PA hydrogels, but it has been difficult to obtain dimensionally stable ultra-soft PDMS materials. In principle, PDMS-based rubbers are readily available as two-component systems and are easily cured under ambient conditions after thorough mixing. Curing is accomplished by platinum-catalyzed hydrosilylation, a polyaddition reaction which implies that no side products are formed. Furthermore, PDMS exhibits virtually no shrinkage upon curing which drastically facilitates the molding procedure. A very important advantage of PDMS over PA hydrogels is the unlimited shelf-life under ambient conditions compared to several days or at best several weeks under refrigeration in the case of PA hydrogels. Sylgard® 184 (Dow Corning) is used as an encapsulant for electronic devices and has been employed to prepare elastic PDMS cell culture substrata [10]. A most recent study suggests that Sylgard® 184-based PDMS substrata fail to induce elasticity-dependent cellular effects [11]. This is in contrast to an earlier study reporting elasticity-dependent cellular effects using this substratum [12]. Unfortunately, commercial two-component PDMS-systems have some disadvantages. Although the hardness of a commercial PDMS-elastomer, determined by the degree of cross-linking, can be adjusted in a certain range by the ratio of the two components, they are not optimized for the preparation of ultra-soft elastomers, i.e. elastomers with Young’s moduli <100 kPa. This fact becomes apparent in the form of a very viscous and sticky material which is obtained when a minimum concentration of the hardening component is used for the curing. Such materials are hardly amenable to further processing steps and we were unable to generate satisfactory ultra-soft cell culture substrata using these systems. Very recently, it was suggested to use blends of two commercially available PDMS types to fabricate cell substrates with an elastic modulus anywhere between 5 kPa and 1.72 MPa [13]. Elastic PDMS substrates were also used in [14] in order to apply mechanical force on neural cells (mechanotransduction).

In all previous works the mechanical properties of PDMS-based cell substrates cannot be changed after fabrication. Magnetoactive elastomers (MAE) [15]–[20] represent a composite material of spherical iron particles embedded in an elastomeric PDMS matrix whose rheological properties are magnetically tunable due to the ferromagnetic properties of the iron. This feature represents the main advantage of MAE over the elastomeric PDMS and PA hydrogels since a single material with a sufficiently low Young’s modulus in the pristine state could yield stiffer substrata in the magnetized state. Hence, a single material might provide different characteristics for attaching cells depending on the magnetic field strength applied. Furthermore, movements of the material can be induced by varying the magnetic field strength or localization. Ultra-soft MAE may thus allow to mimick dynamic biomechanical features and serve to build seals, valves and pumps for biomedical devices. MAE with E-moduli in the MPa range have been developed e.g. to build dynamic damping devices, but inherently stable MAE in the low kPa range have not been reported and no data are available on the use of soft MAE as cell culture substrata.

Therefore, our goals were (1) to generate inherently stable non-viscous PDMS-based cell culture substrata with a Young’s modulus in a biologically relevant range (<100 kPa), which could enhance standard cell culture techniques and (2) to establish compliant MAE cell culture substrata to enable a magnetically tunable elastic modulus and for dynamic mechanotransduction in a cell culture environment.

## Results and Discussion

### Ultra-soft PDMS and MAE Baseline Characteristics

**Figure 1:**
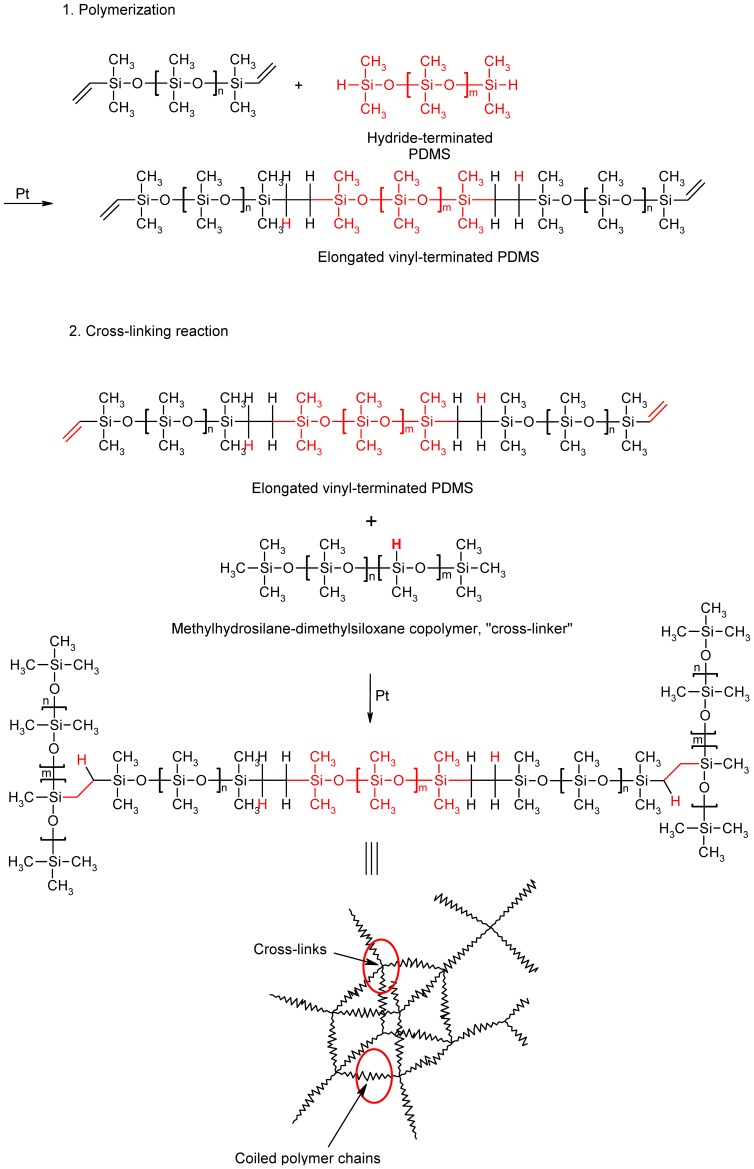
Synthesis of the silicone elastomer matrix. The PDMS is cross-linked via Pt-catalyzed hydrosilylation.

Elastomeric silicone is generally prepared through platinum-catalyzed addition of vinyl-terminated PDMS to a cross-linker, resulting in a comb-like hydride-functionalized PDMS (Fig. 1). The degree of cross-linking is usually controlled by the molecular weight of the starting materials and the molar ratio of vinyl to hydride groups. To guarantee a largely complete reaction of the vinyl groups, a 1.5- to 2-fold excess of hydride groups is commonly applied. In order to achieve soft, extremely elongated polymer structures, a polymerization reaction of the polymeric monomers must be accomplished prior to the crosslinking reaction. This can be managed by the incorporation of hydride-terminated PDMS into the uncured silicone rubber formulation. Since hydride groups at the chain ends are significantly more reactive than those within the polymer chain of the cross-linker, the polymerization reaction takes place preferentially at the chain ends (Fig. 1). As a result, the pot life is also extended significantly. In order to guarantee a complete reaction of the monomers, a large excess of cross-linker with remotely distributed hydride groups was applied (SiH/vinyl ratio of 10).

**Figure 2:**
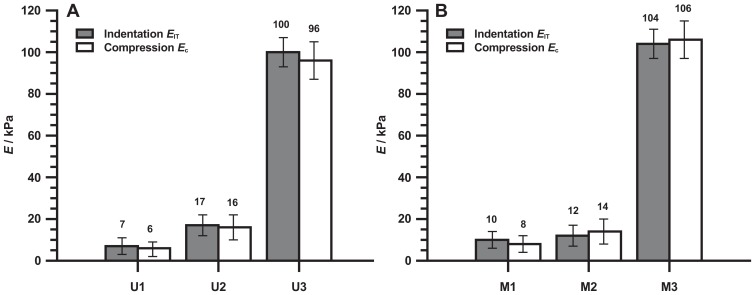
Elastic moduli of the elastomers in the absence of magnetic field as measured by different methods. All indentation data are average values over the entire surface and the indentation depth is 200 µm. (A) Unfilled PDMS matrix; (B) MAE filled with 30 vol. % of CIP. Values represent the average of five separate experiments. Error bars show the standard deviations from the mean.

By hydrolysis of the high molecular cross-linking agent, the number of cross-linking sites is reduced and an excess of silanol groups generated. In this way the reaction sites (OH groups) at the surface of the PDMS are created. The surface is consequently enabled for the bonding of aminopropyl-triethoxysilane (APTES) without plasma treatment. This is advantageous, because the plasma treatment may alter mechanical properties of PDMS surface [21]. These modifications allowed the preparation of dimensionally stable substrata with low Young’s moduli (<20 kPa, Fig. 2A) and surface biocompatibility. MAE as a composite typically exhibit iron loads around 30 Vol.-% corresponding to 77 wt.-%. As a consequence, an elastomeric base formulation undergoes an increase in hardness when large amounts of solid fillers are incorporated. To overcome this problem, further modifications of the silicone rubber were necessary and a baseline elasticity of 14 kPa was accomplished using a plasticizer concentration of 67% by volume. Note that leaching of silicone oil or free polymers can be observed in the PDMS-based elastomers of some compositions [13], [22], [23]. For example, we observed minor leaching of silicone oil in the MAE filled with 30% Vol.-% of carbonyl iron powder (CIP) if the volume concentration of plasticizer exceeded approximately 70%. This concentration of plasticizer is attributed to the percolation threshold of the silicone oil in the MAE composite. This high percolation threshold can be explained by formation of large polymer chains being more capable to bond large amounts of solvent molecules. In all samples presented in this paper no indications of leaching were observed in cell experiments in the time range of up to 14 days.

To avoid iron particle sedimentation during the vulcanization process, a fast reacting room-temperature-vulcanizing Pt-catalyst (Karstedt catalyst) was employed at elevated temperatures. The distribution of iron particles in the elastomer was isotropic. With combined methods it was possible to prepare dimensionally stable substrata with low Young’s moduli (<20 kPa, Fig. 2B) which compare to the hardness of the unfilled silicone substrata. To the best of our knowledge MAE with Young’s moduli lower than 20 kPa and proven bio-compatibility are unprecedented in the literature.

### Magnetic Field Generation for MAE use in Cell Culture Applications

**Figure 3:**
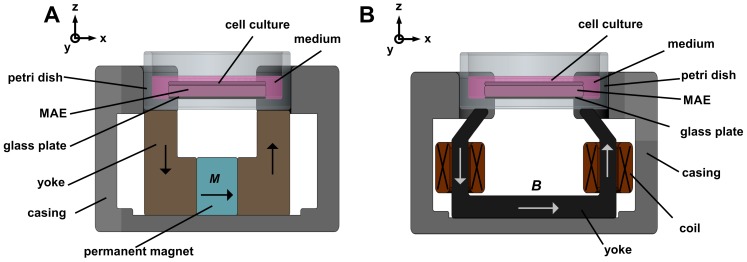
Concepts for controlling the MAE substratum in cell experiments. (A) Schematic of the static device for generating different *E*_IT_ on the MAE surface. (B) Schematic of the dynamic device for introducing displacement field and strain on the surface of the MAE substratum.

We developed devices for the application of magnetic fields to MAE in a standard cell culture environment. The devices are compatible with 24 well cell culture plates or 35 mm Petri dishes, allow placement under an upright microscope and are robust enough to easily withstand 37°C in the humid atmosphere of a cell culture incubator. Fig. 3 illustrates the functional principle for producing static (Fig. 3A) or time varying (Fig. 3B) magnetic fields. The static magnetic circuit consists of two permanent magnets and two magnetic yokes. This circuit guides the magnetic field from the magnetic field sources (permanent magnets) into the MAE samples through the non-magnetic gaps formed by the cell carrier and the glass plate. The dynamic magnetic circuit comprises two current-driven coils rather than a permanent magnet.

#### Bulk properties of MAE in magnetic field

**Figure 4:**
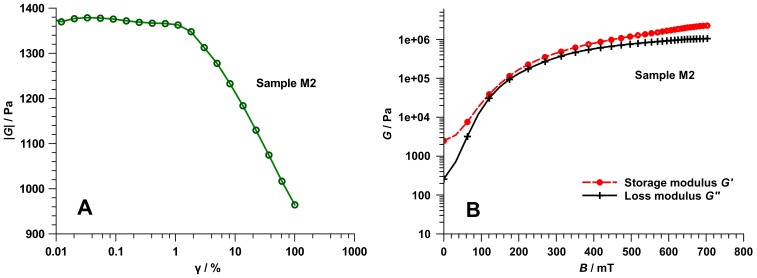
Rheological characterization of the MAE sample M2. (A) Shear modulus |*G*| = |*G*′+*jG′′*| versus shear strain γ (measurement parameters: amplitude sweep, *f = *1 Hz, 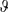 *= *25°C). (B) Complex shear modulus G* versus magnetic flux density *B*_z_ (measurement parameters: *f = *1 Hz, γ = 1%, 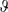 *= *25°C, *F*_N_ = 0.1 N).

The oscillatory shear test is commonly used to characterize the dynamic mechanical properties of MAE. It allows determination of the complex shear modulus *G* = *G*′+ *jG′′* (*j* is the imaginary operator) as a function of an externally applied magnetic field. Fig. 4A illustrates that the shear modulus remains constant for small deformations (*γ*<2%). Fig. 4B shows *G* for an ultra-soft MAE sample M2 and illustrates the ability to change the shear modulus by three orders of magnitude. Low magnetic flux density (*B*∼100 mT) suffices to induce a pronounced increase (more than one order of magnitude) of the shear modulus (Fig. 4B). The maximum magnetic flux density was 0.7 T as determined upon calibration with a Hall probe.

#### Magnetic field-induced elasticity modulation

**Figure 5:**
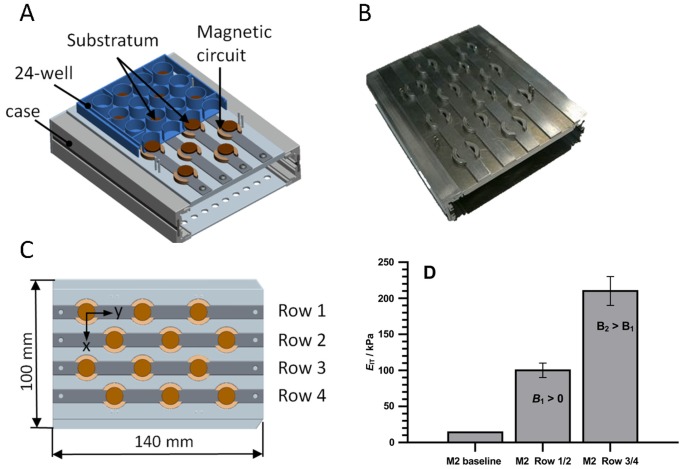
Static device for controlling the indentation modulus *E*_IT_ on the MAE surface (sample M2). (A) Schematic diagram of the device in combination with the well24 cell carrier in a cut illustration. (B) Photograph of the assembled prototype. (C) Dimensions of the device and the positions of the magnetic circuits (top view). (D) *E*_IT_ in the geometrical center of the MAE surface. *E*_IT_ value in the absence of magnetic field (M2 baseline) is shown for comparison. Values represent the average of five separate experiments. Error bars show the standard deviations from the mean. (D) Conditions for the magnetic field: 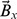 values *B*_1_≈20 mT and *B*_2_≈35 mT (in the geometrical center of MAE surface), Hall probe HMNTAN-DQ 02-TH).

**Figure 6:**
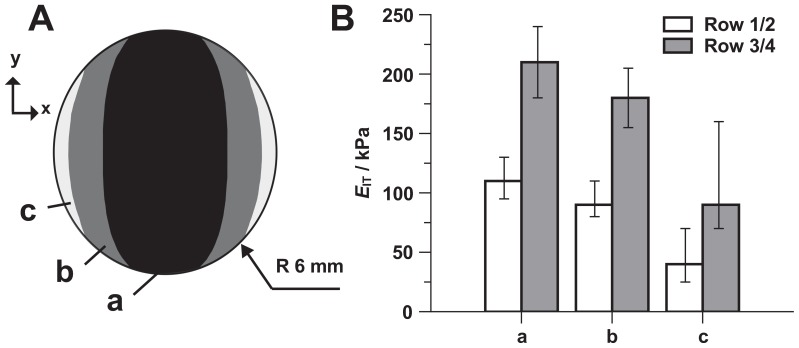
Inhomogeneous distribution of *E*_IT_ over the MAE surface (sample M2). (A) Areas with different levels of *E*_IT_ on the MAE surface. (B) Average values of *E*_IT_ in different areas (rows 1/2 and 3/4). Values represent the average of three separate experiments. Error bars show the range of values measured.

The static device for magnetic field application to MAE in a 24-well cell culture plate and the corresponding values of *E*_IT_ (elasticity modulation) in the center of substratum surface are shown in Fig. 5. The magnetic circuits in row 1/2 & 3/4 generate magnetic fields of two different strengths. The *B*-field in row 3/4 is higher than that in row 1/2, thus the MAE in row 3/4 rows have a higher *E*_IT_ values. Due to the restrictions imposed by the limited sample size and boundary effects, the magnetic field is not entirely homogeneous over the entire substratum. Consequently *E*_IT_ is also inhomogeneous within each 12 mm diameter of an MAE sample. To this end, we performed micro indentation measurements with a penetration depth of 200 µm to obtain the static indentation modulus *E*_IT_, measured in kPa. Fig. 6 illustrates the distribution of the indentation moduli on the surface of the MAE sample M2 (baseline *E*_IT_ = 14 kPa) using a magnetic device as shown in Fig. 5. It may be seen that in the central region of the substratum the elastic properties vary only slightly (±10%).

In the absence of an applied magnetic field, good agreement (discrepancy of about 10% was within the uncertainty of measurements) between indentation test and uniaxial compression test results were found for penetration depths from 180 µm.

#### MAE as actuators in time-varying magnetic fields

**Figure 7:**
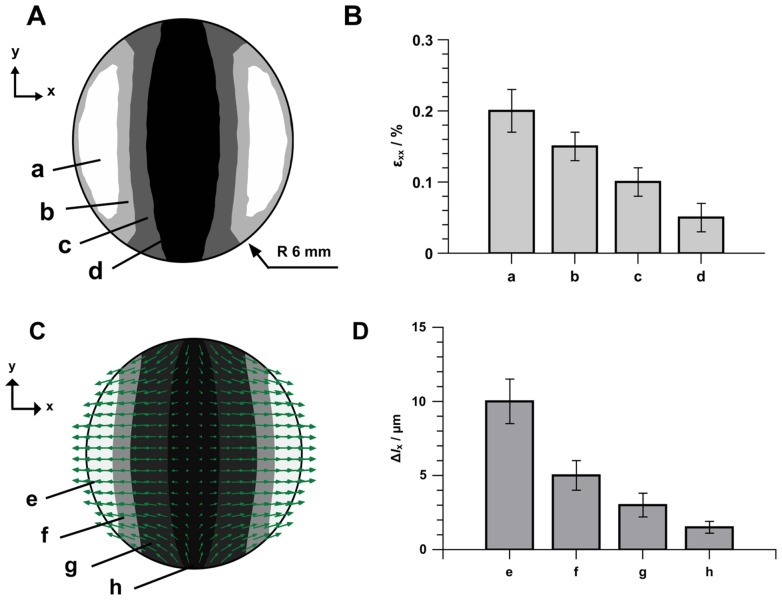
Deformation of the MAE substratum (sample M2) with applied time varying magnetic field. (A) Distribution of engineering strain ε_xx_ = Δ*l*_x_/*l*_x_ at the MAE surface. (B) Average maximum strain ε_xx_ in different areas. (C) Distribution of the displacement vector 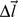 over the MAE surface. (D) Average maximum displacement Δ*l*_x_ in different areas. Values represent the average of three separate experiments. Error bars show the range of values measured. Experiment conditions for the magnetic field: Amplitude 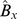 = 10 mT (in the geometrical center of the probe), *f* = 1 Hz, Hall probe HMNA-DQ02-TH. Experiment condition for the recording: Zeiss AxioScope A1, camera AVT Stingray F-125B, frame rate 18 frames/s.

Varying the magnetic fields generated by a magnetic device such as that shown in Fig. 3B induce MAE surface displacements (Fig. 7). The images obtained were analyzed by particle image velocimetry (PIV). Figure 7 shows the results of the analysis which are the displacement vector 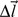 representing the amplitude and the direction of the substratum surface movement at each point on the MAE surface and the strain ε_xx_ = Δ*l*_x_/*l*_x_ along the direction of the applied magnetic field.

### Cell Responses

#### Response to baseline elasticity

To explore whether these novel, soft PDMS-based substrata of different Young’s moduli would result in elasticity-dependent biological effects, we studied smooth muscle actin (α-SMA) expression in human fibroblasts. Earlier observations had established that α-SMA expression decreases in mesenchymal cells with decreasing substratum stiffness [24], [25], [26].

Cells were plated on substrata of different elasticity, allowed to relax for 4–7 days before being harvested for western blot or prepared for immunofluorescence microscopy. Since rigid glass or plastic dishes constitute the current standard substratum for cell culture, glass coverslips were used for control measurements.

**Figure 8:**
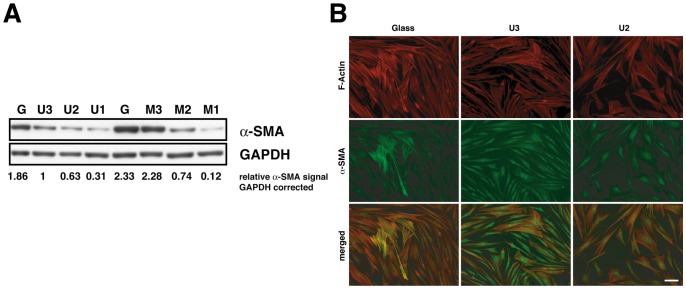
Elasticity-dependent protein expression on soft PDMS (U1–U3) and MAE (M1–M3) substrata in the absence of magnetic field. (A) Western Blot and (B) immunofluorescence analysis show decreased expression of the myofibroblast marker α-smooth muscle actin with decreasing *E*-modulus of the substratum. “G” refers to glass in (A).

Here, we found that the α-SMA expression was lower when cells were cultivated on the newly devised very soft U1/U2 & M1/M2 (<20 kPa) as compared to more rigid PDMS U3 and MAE M3 (120 kPa) substrata and was highest on hard glass. We made similar observations with MAE of comparable pliability (Fig. 8A). Similarly, actin stress fibers were most pronounced in cells on rigid substrata (Fig. 8B). Cells incorporated α-SMA into actin stress fibers on glass and rigid elastomers U3/M3 (100 kPa), but failed to do so on soft elastomers (17 kPa), indicating viable elasticity-dependent regulation. These data indicate that ultra-soft PDMS and PDMS-based MAE exert elasticity-dependent effects on human cell differentiation.

#### Response to magnetic field-induced changes in substratum characteristics

**Figure 9:**
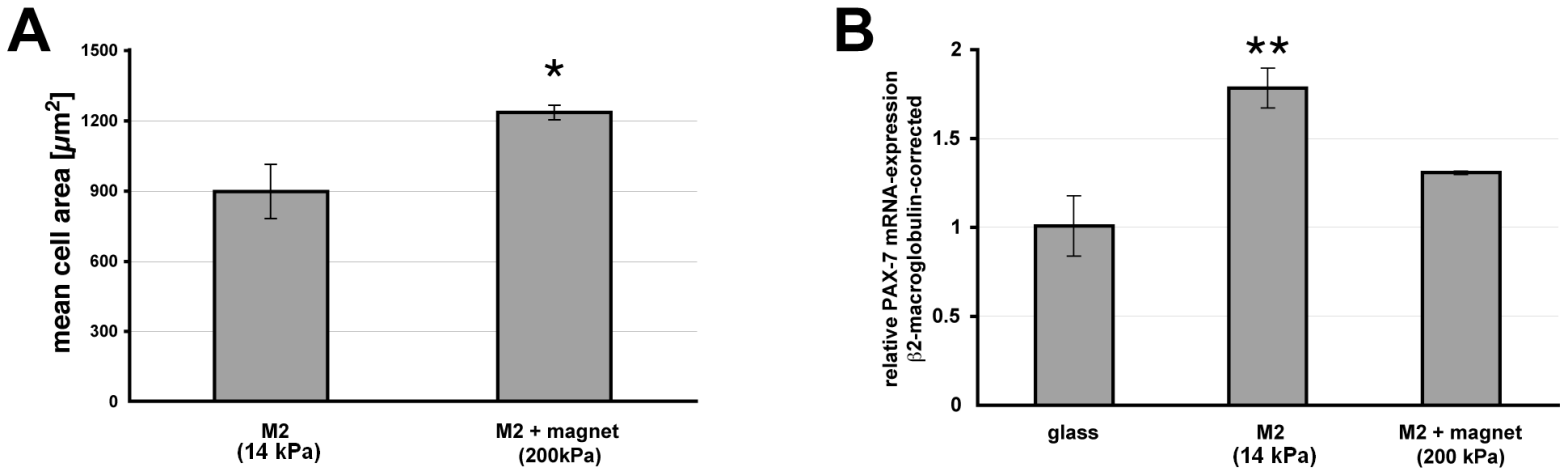
Effects of magnetic field-induced change in substratum properties. (A) Mean fibroblast cell area 60 min after plating increases with substratum E-modulus. (B) Transcription of the muscle satellite cell marker PAX-7 in human mesenchymal stem cells decreases with increasing substratum E-modulus. Triplicate mean ±SEM. Asterisks indicate significance of difference from controls **p<0.01, *p<0.05.

To study possible effects of magnetic field-induced changes in MAE properties on adherent cells, we used a device as characterized in Figure 6 to increase the E-moduli from 14 kPa (M2) to approx. 110 kPa or 210 kPa. While robust changes in α-SMA expression in human fibroblasts were not observed (data not shown) in this system, we detected subtle but significant changes in fibroblast cell spreading and PAX-7 transcription in hMS cells (Fig. 9). Cell spreading is modulated by substratum elasticity [26], [27] as it depends on cell-matrix interactions and cytoskeletal force generation. Stiffening of MAE from approximately 14 to 200 kPa was associated with a 30% increase in mean cell area (895±115 µm^2^ to 1232±31 µm^2^, *p*<0.029, two-tailed student’s t-test, Figure 9A), indicating a significant effect of magnetic field-induced MAE property changes on cell spreading.

Substratum elasticity has also been shown to influence mesenchymal stem cell and myotube differentiation [3], [28]. We therefore studied the effects of magnetic field-induced MAE alterations on protein transcription in human mesenchymal stem cells (Fig. 9B). The transcription factor PAX-7 was transcribed at significantly higher levels (1.78±0.11, *p* = 0.002, two-tailed student’s t-test) on soft MAE (14 kPa, M2) as compared to glass. When MAE were stiffened from 14 to 200 kPa by a weak magnetic field, less PAX-7 mRNA was detected (Fig. 9B, 1.30±0.01, *p* = 0.098, change from control n.s., two-tailed student’s t-test). In line with these findings, the expression of the muscle satellite cell marker PAX-7 has been shown to diminish with increasing substratum stiffness in hydrogel systems [29]. It is currently unclear why the cell response to magnetic field-induced changes is not entirely similar to effects of different baseline elasticity in MAE. However, magnetic fields alter several mechanical characteristics of MAE simultaneously, e.g. plasticity increases with field strength as elasticity decreases. Changes in surface structure due to field-alignment of magnetic particles may also occur and modulate cell adhesions. On the other hand, less pronounced effects of magnetic field-induced MAE changes on protein expression may prove advantageous as this may facilitate the exploitation of MAE in cell-coated actuators in biocompatible devices.

**Figure 10:**
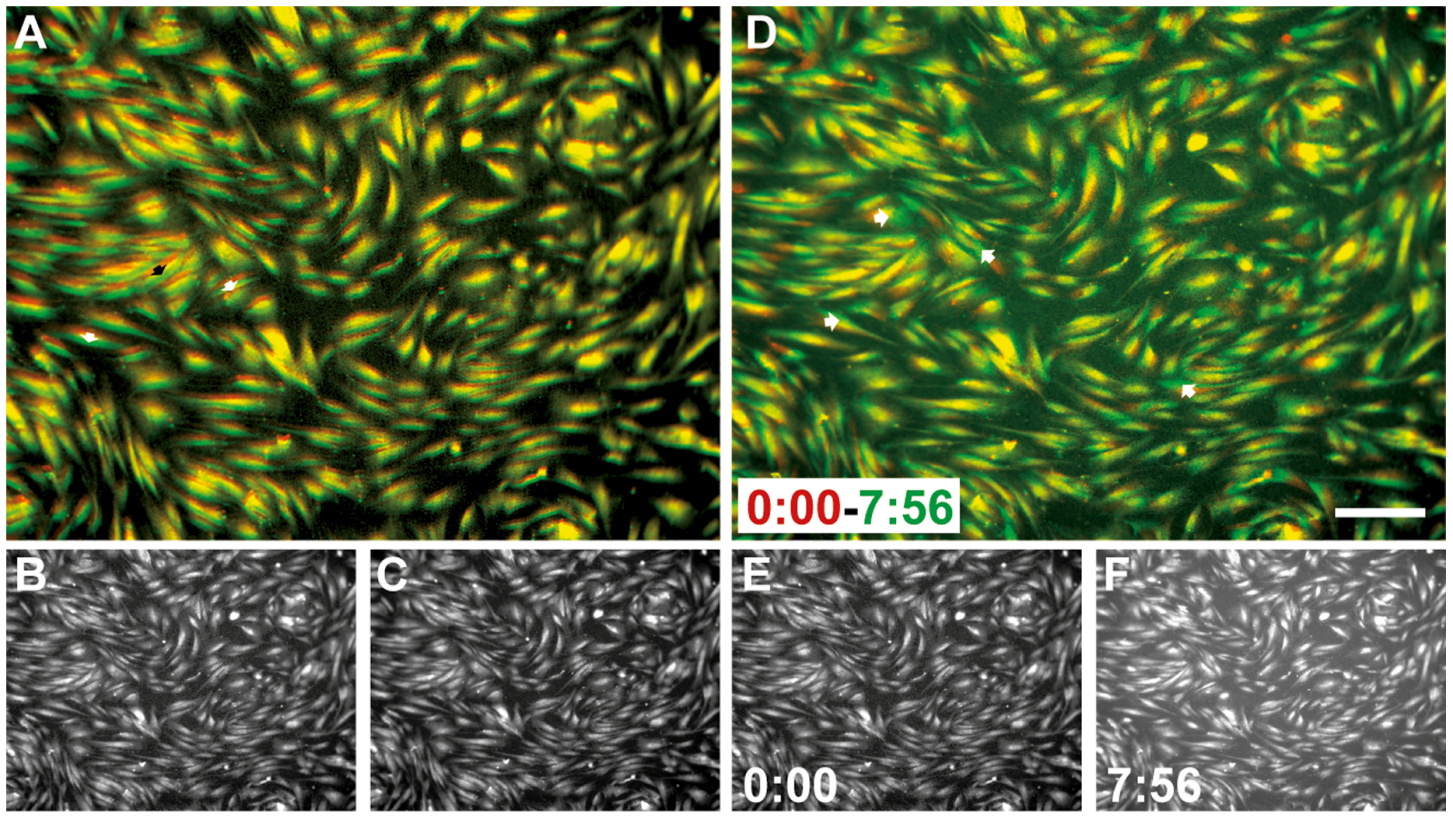
MAE movement (*f* = 1 Hz) by varying magnetic field strength is well tolerated by attached human dermal fibroblasts. (A) Composite image of (B, red and C, green) depicting displacement extremes. (D) Cell movement in 7 h: 56 min. Composite image of first (E, red in D) and last frame (F, green in D) of a time lapse series with 15 frames/h.

Magnetic fields of time-dependent strength can induce displacements in the MAE surface. In the pilot systems used, the movements were anisometric (Fig. 10) and difficult to predict. However, it was necessary to evaluate the principal biocompatibility of a dynamic MAE-based stimulation configuration. Primary human fibroblasts were stained with CMTR cytotracker dye to assist visualization before being applied to soft MAE (14 kPa, M2) surfaces. Following a 24 h stabilization period they were subjected to 1 Hz magnetic field stimulation which induced pulsatile surface translocation (Fig. 10A–C). Time lapse observations over several hours revealed intact cell migration without significant cell detachment (Fig. 10D–F).

Ultra-soft MAE may be used to generate cell-coated valves or pump systems actuated by magnetic field generators. Furthermore, actuated cell culture substrata may allow to improve the cultivation of specific cell types e.g. to generate muscle or tendon constructs in vitro. To this end, modifications of surface topography or composite constructs with hydrogel surfaces offer additional possibilities.

## Conclusions

Our data indicate that dimensionally stable, PDMS-based cell culture substrata with *E*-moduli <20 kPa can be produced by using appropriate crosslinkers and high molecular weight PDMS monomers. The PDMS-based materials elicited protein expression changes typically observed with soft cell culture substrata and may thus serve to improve current standard cell culture hardware. It is also possible to obtain MAE with similar characteristics. Magnetic fields can be employed to modulate biomechanical properties of very soft MAE to alter distinct cell functions and to induce surface translocations. These observations suggest that cell sheet-coated MAE may form the basis of a whole range of novel actuators suitable for the design of future biomedical devices.

## Materials and Methods

### Material Composition and Preparation

Vinyl-terminated polydimethylsiloxane can be purchased either from Hanse Chemie or Gelest and Hydride functional silane from Hanse Chemie. The Karstedt catalyst was purchased from Gelest. The plasticizer, low molecule weight silicone oil AK10, was obtained from Wacker Chemie and the carbonyl iron powder purchased from BASF (type SQ, mean diameter of 4,5 µm). All chemicals were used without further purification. The MAE substrates were prepared by the cross-linking of a liquid silicone rubber dispersion containing 30% of CIP by volume.

After thorough compounding using a speed mixer (Hausschild DAC 150.1 FVZ) for 3 minutes at 2500 RPM and removal of air in vacuum (20 min), curing was completed after only one hour at 100°C. Due to the high reactivity of the catalyst system a platinum concentration of 10 ppm was sufficient to accomplish the vulcanization process completely. In order to obtain precisely shaped MAE sheets, high-quality PTFE-coated tools (45×45×2) mm, were used for molding. Finally, the test specimen for tissue cultivation, (12×2) mm, compression test (20×6) mm and rheological characterization, (20×2) mm, was simply die cut.

Three different base elastomers of PDMS/MAE samples were prepared during this work. (cf. Figure 2). Furthermore the mechanical properties of corresponding samples U1/M1, U2/M2, U3/M3 exhibited approximately the same values (within the accuracy of measurements). This was achieved by varying the elastomer components. The unfilled samples U1 and U2 differ only in the amount of plasticizer used which is 90 and 78 Vol.-%, respectively. The MAE M1 and M2, differ as well only by the concentration of plasticizer, which are approximately 67 Vol.-% for M1 and 60 Vol.-% for M2, respectively.

The silicone matrix of U3 and of the MAE sample M3 differ only by the type of cross-linker applied. The ratio of vinyl-polymer to chain extender and the amount of plasticizer were maintained constant. The MAE samples M3 utilized a high-molecular weight cross-linker whereas a comparatively low-molecular-weight cross-linker was applied in the unfilled sample U3. This has been done in order to accommodate for the stiffening of MAE samples due to the presence of CIP.

### Characterization of Mechanical Material Properties

Three different methods were used to measure the mechanical properties of PDMS-based elastomers. Two of them, namely oscillatory shear test (OST) [30] and uniaxial compression test (UCT) [31], [32] are used to determine the mechanical properties of bulk viscoelastic samples. The result of OST is the complex shear modulus *G* = *G*′+*jG*′′ as a function of the externally applied magnetic flux density *B*. This is a conventional dynamic characterization technique for MREs [18], [17], [33]–[35]. The result of UCT is the static (compression) Young’s modulus *E*_c_. It was not possible to perform the UCT with the applied magnetic field. In addition, UCT and OST cannot be used to measure local mechanical properties of elastomers. Therefore a conventional micro-hardness tester (MHT) [36], [37] was used for these purposes. Local measurements were needed because of the inhomogeneous distribution of the magnetic field over the sample’s surface (cf. Fig. 6). Although *E*_IT_≈*E*_c_ they are not exactly the same (cf. Fig. 2) property. MHT describes the stiffness behavior only locally and under triaxial stress. This creates a difference to the elastic modulus *E*_c_ determined by conventional UCT. The results of OST and UCT/MHT cannot be directly compared because OST is a dynamic test while the other two methods (UCT/MHT) are static tests.

The rheological characterization was carried out on a MCR 501 rheometer from Anton Paar, Austria. The shear modulus *G* as a function of the magnetic flux density was measured in oscillation mode using a plate/plate system (measurement parameters: *f = *1 Hz, *γ = *1%, *θ = *25°C, *F*_N_ = 0.1N).

To locally examine the mechanical properties of the MAE surface, conventional micro-hardness measurement by the so-called penetration method was used. In this case a Vickers pyramid shaped diamond head penetrates to a depth of 200 µm into the sample surface which barely damages the sample. The automated surface characterization has been performed using a FISCHERSCOPE® HM2000 device with the corresponding software package WIN-HCU® 4.4 supplied by Helmut Fischer GmbH, Sindelfingen, Germany. The measurement parameters using an indenter type H2N 17201110 were as follows: test load 15–20 mN, application duration 20 s. The Poisson’s ratio for MAE samples has been estimated from the literature to *υ*≈0.49 [38]. The result of interest is the indentation *E*_IT_. This material parameter was analyzed by the unloading curve of the MHT, according to the standard DIN ISO 14577 [39]. The theory of this method (Oliver Pharr method) for the determination of *E*_IT_ is described in [40].

Moreover the indentation device was complemented by calibrated magnetic circuits (cf. Fig. 3A) allowing measurements to be conducted with an applied magnetic field.

The static magnetic device in Figure 5D generate a *B* field in the x direction of *B*_1_≈20 mT (Row 1/2) and *B*_2_≈35 mT (Row 3/4) in the geometrical center of the MAE substratum (M2, 14 kPa). This value was measured using a gaussmeter (Lakeshore 455 DSP, Hall probe: HMNA-DQ02-TH). In order to determine the average values of *E*_IT_ in Figure 6 the MHT measurements were performed on a number of points over the entire surface of the MAE substratum. Since the edge regions are hardly accessible with the indenter, the measured values of the indentation modulus were extrapolated into the boundary area.

### Characterization of MAE Surface Deformation

The deformations of the MAE surface (Fig. 7) were analyzed with the method of PIV [41], [42]. Initially, the substratum surface was functionalized with fluorescent particles having an average diameter of 3 µm, obtained from MR® Chemie GmbH (Unna, Germany). In a second step MAE samples were excited with a time-dependent magnetic field as shown in Figure 4B. The amplitude (sinusoidal form) of magnetic flux density was 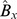 = 10 mT at a frequency of *f* = 1 Hz. This was measured with a gaussmeter (Lakeshore 455 DSP, Hall probe: HMNA-DQ02-TH) in the geometrical center and top of the sample.

In the final step image sequences centered at several points equally distributed over the entire MAE surface were digitally recorded using the fluorescence microscope (Zeiss AxioScope.A1, camera AVT Stingray F-125B, frame rate 18 frames/s). The visibility field of the microscope was about 1.7×1.3 mm. The images were processed according to the conventional PIV procedure using JPIV [43] software to calculate the displacement vector 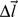 and the engineering strain ε_xx_ = Δ*l*_x_/*l*_x_ on the MAE surface. The displacement vector describes the difference between a pixel (fluorescent particle on the MAE surface) in the final position (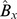 = 10 mT) with reference to the initial position (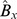 = 0, time *t = *0). Additionally, the engineering strain (of a small deformation, Δ*l*_x_ = 2 pixel distance) ε_xx_ = Δ*l*_x_/*l*_x_ describes the relative rate of displacement changes on the MAE surface (displacement gradient). The displacement vector 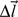 at intermediate positions and the strain ε_xx_ were obtained by interpolation.

### Cells and Substratum Preparation

Human dermal fibroblasts were obtained from Provitro (Berlin, Germany) and human mesenchymal stem cells from Lonza (Basel, Switzerland). Cells were cultured in Dulbeccós Modified Eagle’s Medium (DMEM, PAA Laboratories GmbH; Pasching, Austria) supplemented with 10% heat inactivated fetal calf serum (FCS, Biochrom, Berlin, Germany), 100 U/ml penicillin and 100 µg/ml streptomycin (both from PAA) as suggested by the supplier and used in passages 3–10. Experiments were performed at least three times with similar results. To provide ECM coating for cell attachment, PDMS and MAE surfaces were treated as previously described [44] with slight modifications. In brief, surfaces were silanized with 2% Aminopropyl-triethoxysilane (APTES) for 15 min, washed extensively and subsequently treated with 0.25% glutardialdehyde for 20 min, washed, coated with fibronectin (5 µg/ml), blocked with 0.1% heat-denatured BSA and washed in PBS. Circular PDMS or MAE samples of 12 mm diameter and 2 mm thickness on a 12 mm round coverslip were used in 24 well plates for expression analysis or in 35 mm dishes for life cell microscopy.

### Western Blot

Cells were serum-starved for 16 h, plated on the substrata and allowed to adjust for 5 days. Cells were rinsed with cold (4°C) PBS and total cell protein extracts were prepared using a RIPA lysis buffer (20 mM TRIS, 150 mM NaCl, 0.1 mM EDTA, 1% Triton X-100, 1% Deoxycholate, 0.1% SDS) containing phosphatase and protease inhibitors (Phosphatase Inhibitor Cocktail III, Calbiochem/Merck, Bad Soden, Germany; Complete Protease Inhibitor, Roche, Mannheim, Germany). Protein extracts were boiled in Laemmli sample buffer, subjected to SDS polyacrylamide gel electrophoresis and transferred onto a PVDF membrane (Amersham, Braunschweig, Germany) using a BioRad gel blotting apparatus. Membranes were blocked in 3% BSA in TBST (10 mM TrisHCl, 150 mM NaCl, 0.1% Tween 20) for 1 hour. Membranes were incubated with primary antibody to α-SMA (Sigma, Schnelldorf) overnight at 4°C and with a peroxidase-conjugated secondary antibody (Jackson Immuno Research, Newmarket, UK) for 60 min at room temperature. After each incubation step, membranes were washed in TBST for 30 min. Peroxidase was visualized by Enhanced Chemoluminescence and exposure to Hyperfilm ECL films (both Amersham, Braunschweig, Germany) for appropriate times.

### qPCR

Cells were rinsed with PBS, gently scraped off the substrate and collected by centrifugation. The cell pellet was then processed using the RNeasy kit (Qiagen, Hilden, Germany) as recommended by the manufacturer. Two µg of extracted RNA were reverse transcribed (Superscript II, Qiagen) using Oligo-dT primers (Promega). A commercially available kit (SYBR Premix Ex Taq II, Takara Bio Inc., Otsu, Japan) was used for SYBR-green-monitored real-time PCR amplification performed in triplicates on a Step One plus cycler (Applied Biosystems, Foster City, U.S.A.). Primers were: β-Macroglobuline (left: TATCCAGCGTACTCCAAAGA, right: GACAAGTCRGAATGCTCCAC), PAX-7 (left: CACTGTACCGAAGCACTGT, right: TTCTTGTCCGCTTCATCCTC). Enzyme activation (95°C, 20 s) was followed by 40 cycles of denaturation (95°C, 5 s), primer annealing (54°C, 10 s) and primer extension (60°C, 20 s). mRNA levels were determined as CT threshold levels and normalized with the individual β-Macroglobuline control CT values. CT cycle number differences between unstimulated expression levels on tissue culture plastic (thus equal to 1) and the respective condition were calculated. Its power of two represents the relative mRNA level which is presented as mean ± SEM± of triplicate analyses. Two-tailed students t-test was used for statistical analysis.

### Cell Spreading

Human dermal fibroblasts were trypsinized, maintained in suspension in a cell culture incubator for 1 hour to allow for equal retraction of all cells and subsequently plated on collagen-coated substrata. 40 min after plating the cells were fixed in 2% paraformaldehyde (Merck, Mannheim, Germany), permeabilized with 0.1% Triton X100 and F-actin was stained with Phalloidin-TRITC (Sigma). After washing in PBS the stained samples were mounted in Vectashield (Vector, Burlingame, U.S.A.) and viewed with a fluorescence microscope (Axio, Zeiss, Oberkochen, Germany). To assess cell spreading, slide labels were blinded and cell area was measured in all cells of three random fields capturing at least 30 cells using NIH-image software. The groups were analyzed in a two-tailed students t-test.

### Time Lapse Imaging of Cells on Actuated MAE

Dermal human fibroblasts were fluorescently labeled using CMTMR celltracker dye following the manufacturer’s instructions (Invitrogen), plated on fibronectin-coated MAE and allowed to spread overnight. Next, the cells were transferred to L - 15 medium (PAA, Pasching, Austria) containing 10% FCS and mounted on the custom-built magnetic field stimulator (Fig. 3A) under an upright fluorescence microscope (Axio, Zeiss, Oberkochen, Germany) with a custom-built shutter and automated camera. Water immersion lenses were used to image the labeled cells on the opaque MAE substrata. This simple setup allowed the recording of timelapse series for several hours.
